# LDB1 Is Required for the Early Development of the Dorsal Telencephalon and the Thalamus

**DOI:** 10.1523/ENEURO.0356-18.2019

**Published:** 2019-03-12

**Authors:** Veena Kinare, Suranjana Pal, Shubha Tole

**Affiliations:** 1 Department of Life Sciences, Sophia College for Women, Mumbai 400026, India; 2 Department of Biological Sciences, Tata Institute of Fundamental Research, Mumbai 400005, India

**Keywords:** Cre recombinase activity, forebrain, inefficient floxing, Ldb1, somatosensory thalamus

## Abstract

LIM domain binding protein 1 (LDB1) is a protein cofactor that participates in several multiprotein complexes with transcription factors that regulate mouse forebrain development. Since *Ldb1* null mutants display early embryonic lethality, we used a conditional knockout strategy to examine the role of LDB1 in early forebrain development using multiple Cre lines. Loss of *Ldb1* from E8.75 using Foxg1Cre caused a disruption of midline boundary structures in the dorsal telencephalon. While this Cre line gave the expected pattern of recombination of the floxed *Ldb1* locus, unexpectedly, standard Cre lines that act from embryonic day (E)10.5 (Emx1Cre) and E11.5 (NesCre) did not show efficient or complete recombination in the dorsal telencephalon by E12.5. Intriguingly, this effect was specific to the *Ldb1* floxed allele, since three other lines including floxed Ai9 and mTmG reporters, and a floxed *Lhx2* line, each displayed the expected spatial patterns of recombination. Furthermore, the incomplete recombination of the floxed *Ldb1* locus using NesCre was limited to the dorsal telencephalon, while the ventral telencephalon and the diencephalon displayed the expected loss of *Ldb1*. This permitted us to examine the requirement for LDB1 in the development of the thalamus in a context wherein the cortex continued to express *Ldb1*. We report that the somatosensory VB nucleus is profoundly shrunken upon loss of LDB1. Our findings highlight the unusual nature of the *Ldb1* locus in terms of recombination efficiency, and also report a novel role for LDB1 during the development of the thalamus.

## Significance Statement

The role of transcriptional co-factor LIM domain binding protein 1 (LDB1) in mouse forebrain development was examined using a floxed *Ldb1* line and standard Cre driver lines Foxg1Cre, Emx1Cre, and NesCre. Foxg1Cre revealed that LDB1 is a key regulator of early telencephalic midline development. Curiously, the floxed *Ldb1* locus appeared to be selectively resistant to Cre-mediated recombination in the dorsal telencephalon using Emx1Cre and NesCre. Recombination improved with time in the case of Emx1Cre. NesCre recombined the floxed Ldb1 locus efficiently in the ventral telencephalon and in the diencephalon, where a critical requirement for this factor in the development of the somatosensory VB nucleus of the thalamus was revealed. Our findings highlight the importance of assessing the extent of recombination when interpreting conditional loss-of-function phenotypes.

## Introduction

LIM domain binding protein 1 (LDB1; also called NLI/CLIM2) is a protein cofactor with an impressive list of binding partners, and is found in multiprotein complexes in several systems including the nervous system and the hematopoietic system, associated with transcription factors of the GATA, bHLH, LIM-HD, and OTX families (Hobert and Westphal, 2000; Matthews and Visvader, 2003; Love et al., 2014). LDB1 has domains that interact with its partners, and also a dimerization domain that permits it to serve as a “bridge,” bringing together multi-protein complexes associated with each LDB1 monomer (Jurata and Gill, 1997; Milán and Cohen, 1999; Van Meyel et al., 1999, 2000; Thaler et al., 2002). LDB1, therefore, functions as a nuclear adaptor forming tetrameric, hexameric, or other higher-order protein complexes with these transcription factors, thereby enabling their function (Matthews and Visvader, 2003; Love et al., 2014).

Murine *Ldb1* is widely expressed in the entire embryo starting from the earliest stages of development (Bach et al., 1997; Visvader et al., 1997; Mukhopadhyay et al., 2003). *Ldb1* null mutants die between embryonic day (E)9.5 and E10.5 and display truncated anterior head structures and loss of heart and foregut formation, indicating a crucial early role in developmental processes (Mukhopadhyay et al., 2003). *Ldb2*, a related family member found in several vertebrate species (Agulnick et al., 1996; Bach et al., 1997; Matthews and Visvader, 2003), has a more restricted expression in the developing brain, limited to the cortical hem and antihem at E12.5, and is expressed in layer 5 neurons starting from late embryonic stages (Bach et al., 1997; Bulchand et al., 2003), where it regulates aspects of corticospinal motor neuron differentiation together with *Ldb1* (Leone et al., 2017).

Of the transcription factors that interact with LDB1, LIM-HD proteins LHX2 and LHX5 play crucial roles in regulating early dorsal telencephalic development. LHX2 has been identified as a cortical selector. *Lhx2* null mutants lack both hippocampus and neocortex, while the non-cortical fates of hem and antihem are expanded (Bulchand et al., 2001; Mangale et al., 2008). LHX5 is critical for medial telencephalic patterning, which is grossly disrupted in *Lhx5* null mutants (Zhao et al., 1999). Another transcription factor, OTX2, is required for the normal development and maintenance of the choroid plexus (Johansson et al., 2013) and is also known to act in a complex with LDB1 (Costello et al., 2015). These studies strongly motivate an examination of whether the functions of these genes in regulating the early development of the cortical primordium require LDB1.

We used a Foxg1Cre driver and a floxed *Ldb1* line to examine the early stages of dorsal telencephalic patterning, and discovered specific deficits in telencephalic midline formation. Using a NestinCre (NesCre) line, we also discovered severe and specific defects in the thalamus in the absence of *Ldb1*. Finally, our data identified an apparent resistance to recombination of the floxed *Ldb1* allele that is selective to the dorsal telencephalon when either NesCre or Emx1Cre is used. Therefore, this study not only identifies novel functions of LDB1 in early forebrain development, but also brings out a hitherto unreported shortcoming of conditional gene deletion strategies using standard Cre lines.

## Materials and Methods

### Mice

All animal protocols were approved by the Institutional Animal Ethics Committee according to regulations devised by the Committee for the Purpose of Control and Supervision of Experiments on Animals. The Foxg1Cre line was obtained from Susan McConnell (Hebert and McConnell, 2000). Mice carrying the floxed *Ldb1* line were obtained from Paul Love, NIH, and Susan McConnell, Stanford, with the kind permission of Yangu Zhao, NIH. Noon of the day of vaginal plug was designated as E0.5. Mouse embryos of either sex were harvested at E12.5, E15.5, E17.5, and postnatal day (P)0. Controls used for each experiment were age-matched littermates. ISH for each marker was performed in more than or equal to three biological replicates. Some of the embryos examined were also heterozygous for the *Ldb2* null allele, but these were indistinguishable from embryos that carried both wild-type alleles, likely due to the very limited expression of *Ldb2* in the brain (Bulchand et al., 2003; Leone et al., 2017).

The Emx1Cre line (strain name: B6.Cg-Emx1tm1(cre)Krj/J; stock number: 005628; Gorski et al., 2002) is the same as the Emx1Cre^KJ^ in Shetty et al. (2013). The NestinCre line is from JAX labs (strain name: B6N.Cg-Tg(Nes-cre)1Kln/CjDswJ; stock number: 019103). The GFP reporter mTmG (strain name: Gt(ROSA)26Sortm4(ACTB-tdTomato,-EGFP)Luo; stock number: 007676) and Ai9 (strain name: B6.Cg-Gt(ROSA)26Sortm9(CAG-tdTomato)Hze/J; stock number: 007909) reporter lines were also obtained from JAX labs. The floxed *Lhx2* line used in this study was obtained from Edwin Monuki, University of California (Mangale et al., 2008).

**Figure 1.**
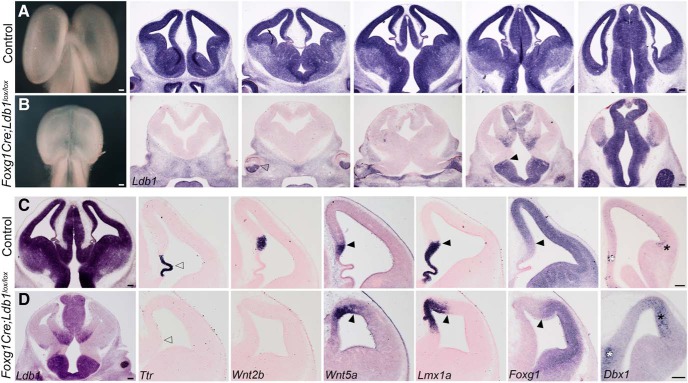
Early deletion of Ldb1 using Foxg1Cre results in reduced telencephalic size and disrupted telencephalic midline patterning. ***A***, ***B***, Whole brains and coronal sections at E12.5 from control (***A***) and *Foxg1Cre;Ldb1^lox/lox^* mutants (***B***) reveal a greatly reduced telencephalon on loss of *Ldb1*. *Ldb1* is expressed in the entire control telencephalon and thalamus at E12.5 (***A***) but is undetectable in the mutant telencephalon (***B***). The expression boundaries of *Ldb1* in the retina (open arrowhead, ***B***) and the diencephalon (black arrowhead, ***B***) are consistent with the reported activity of Foxg1Cre (Hebert and McConnell, 2000). ***C***, ***D***, E12.5 sections from control (***C***) and *Foxg1Cre;Ldb1^lox/lox^* mutant (***D***) brains. The expression of choroid plexus marker *Ttr* is lost in the mutant (open arrowheads, ***C***, ***D***). The mutant does not express hem marker *Wnt2b* but displays an expanded expression of *Wnt5a* and *Lmx1a*. *Lmx1a* labels both hem and choroid plexus in control sections. *Foxg1* is expressed in a manner complementary to *Lmx1a* in control and mutant sections. Black arrowheads mark the cortex-hem boundary (***C***, ***D***). *Dbx1* is expressed in the septum (white asterisks) and antihem (black asterisks), both of which are expanded on loss of LDB1 (***C***, ***D***). Scale bars: 100 μm.

Embryos used for sections in Figures 1, 3–6 did not carry any reporter allele.

Genotyping of the various alleles was performed by PCR using the following primers:

Ldb1-F: 5′-CTTATGTGACCACAGCCATGCATGCATGTG-3′

Ldb1-R: 5′-CAGCAAACGGAGGAAACGGAAGATGTCAG-3′

Foxg1Cre-F: 5’AGTATTGTTTTGCCAAGTTCTAAT-3′

Foxg1Cre-R: 5’TCCTATAAGTTGAATGGTATTTTG-3′

Emx1Cre-F: 5′-ATTTGCCTGCATTACCGGTC-3′

Emx1Cre-R: 5′-ATCAACGTTTTCTTTTCGG-3′

NestinCre-F: 5′-ATTTGCCTGCATTACCGGTC-3′

NestinCre-R: 5′-ATCAACGTTTTCTTTTCGG-3′

Lhx2-F: 5′-ACCGGTGGAGGAAGACTTTT-3′

Lhx2-R: 5′-CAGCGGTTAAGTATTGGGACA-3′

### Sample preparation and *in situ* hybridization

For *in situ* hybridization, the mouse embryos were harvested in PBS, fixed in 4% paraformaldehyde (PFA), equilibrated in 30% sucrose made in 4% PFA, and sectioned at 16 μm on a freezing microtome.

*In situ* hybridization was performed as follows: the sections were fixed in 4% PFA, washed in PBS, and treated with proteinase K (1 µg/ml). Hybridization was performed overnight at 70°C in hybridization buffer (4× SSC, 50% formamide, and 10% SDS) containing different antisense RNA probes. Post-hybridization washes were performed at 70°C in solution X (2× SSC, 50% formamide, and 1% SDS). These were followed by washes in 2× SSC, 0.2× SSC, and then Tris-buffered saline–1% Tween 20 (TBST). The sections were incubated in anti-digoxigenin Fab fragments (Roche) at 1:5000 in TBST overnight at 4°C. The color reaction was performed using NBT/BCIP (Roche) in NTMT [100 mM NaCl, 100 mM Tris (pH 9.5), 50 mM MgCl_2_, and 1% Tween 20] according to the manufacturer’s instructions.

### Probe preparation

All probes were prepared by *in vitro* transcription using a kit from Roche as per manufacturer’s instructions. Templates for *Ldb1 exon1-9*, *Lhx2 exon2-3*, *SERT*, *Chst2*, and *Prox1* were generated by PCR using specific primers from E15 (for *Ldb1 exon 1-9* and *Lhx2 exon2-3*) and P7 (for *SERT*, *Chst2*, and *Prox1*) mouse cDNA (T7 polymerase promoter sequence was added to the reverse primer sequence). Templates for the other probes were generated from respective plasmid DNA by restriction enzyme digestion. Plasmids used were kind gifts from Forbes D. Porter (*Lhx2*), Elizabeth Grove (*Lhx9, Neurog2*), Alessandra Pierani (*Dbx1*), Cliff Ragsdale (*Wnt2b*), Kathleen Millen (*Lmx1a*), and Eseng Lai (*Foxg1*).

Primers for PCR-generated probes:

Ldb1 exon1-9-F: TACCCACCTACATACCTGGA

Ldb1 exon1-9-R: TGAGAGTGGAATTGGACAGC

Lhx2 exon2-3-F: CGCGGATCCACCATGCCGTCCATCAGC

Lhx2 exon2-3-R: TAATACGACTCACTATAGGG

SERT-F: CAAAACGTCTGGCAAGGTGG

SERT-R: CATACGCCCCTCCTGATGTC

Chst2-F: CATCTTTGGGGCAGCCACTA

Chst2-R: CGAAAGGCTTGGAGGAGGAG

Prox1-F: GCAGGCCTACTATGAGCCAG

Prox1-R: TTTGACCACCGTGTCCACAA

## Results

*Ldb1* is ubiquitously expressed throughout the embryonic forebrain (Bulchand et al., 2003). We used three standard Cre driver lines to examine the stage-wise roles of LDB1 in early forebrain development, Foxg1Cre, Emx1Cre, and NesCre. Foxg1Cre acts from E8.75 in the entire telencephalon and in a limited portion of the diencephalon (Hébert and McConnell, 2000). Emx1Cre action is specific to the dorsal telencephalon, and complete recombination is expected from E10.5 (Gorski et al., 2002). NesCre activity initiates a day later, from E11.5, and is active in radial glia in the entire central nervous system (Tronche et al., 1999). We generated a set of male mice each carrying a particular Cre driver and at least one *Ldb1* floxed allele, and crossed them with *Ldb1^lox/lox^* or *Ldb1^lox/lox^*;*Ai9* females.

Intact brains from E12.5 *Foxg1Cre;Ldb1^lox/lox^* embryos appeared distinctly smaller, with a poorly developed midline, compared to controls (Fig. 1*A*,*B*). We examined *Ldb1* mRNA expression in these brains in a series of rostro-caudal sections, and observed the expected, well-characterized pattern of recombination for Foxg1Cre (Hébert and McConnell, 2000), such that the entire telencephalon did not display detectable *Ldb1* expression, but most of the diencephalon and the ventral half of the retina was spared (Fig. 1*A*,*B*). In coronal sections, the midline deficits in *Foxg1Cre;Ldb1^lox/lox^*embryos were obvious (Fig. 1*C*,*D*). The choroid plexus appeared to be missing, both in terms of morphology and *Ttr* expression (open arrowheads; Fig. 1*C*,*D*). The cortical hem lacked *Wnt2b* expression, but the expression of two other hem-specific genes, *Wnt5a* and *Lmx1a*, appeared expanded, and *Foxg1*, a known suppressor of hem fate (Muzio and Mallamaci, 2005; Godbole et al., 2017), was excluded from this region (black arrowheads; Fig. 1*C*,*D*). The septum (white asterisk) and the antihem (black asterisk), both identified by *Dbx1* expression, also appeared expanded in the *Foxg1Cre;Ldb1^lox/lox^* mutant.

**Figure 2.**
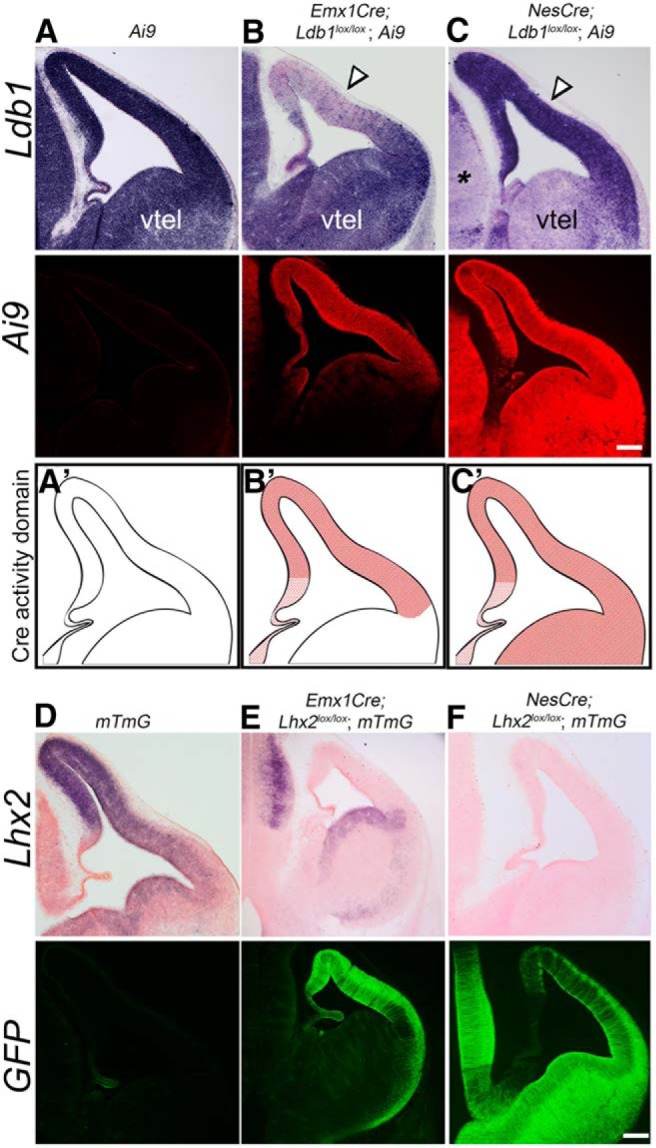
Two standard Cre drivers display differential recombination of the *Ldb1* locus in the dorsal telencephalon. ***A–C***, Expression of *Ldb1* mRNA and the Ai9 reporter in E12.5 control (***A***), *Emx1Cre;Ldb1^lox/lox^;Ai9* (***B***), and *NesCre;Ldb1^lox/lox^;Ai9* (***C***) brains. ***A’–C’***, Cartoons indicating the domain of Cre activity in the respective conditions. In each condition, Ai9 fluorescence faithfully reports Cre activity in the expected domains. However, *Ldb1* expression is seen in a medio-lateral gradient in the dorsal telencephalon of *Emx1Cre;Ldb1^lox/lox^;Ai9* embryos (***B***) and persists in the entire dorsal telencephalon in *NesCre;Ldb1^lox/lox^;Ai9* brains (***C***). In contrast, the ventral telencephalon (vtel) and the diencephalon (black asterisk) display the expected loss of *Ldb1* expression. ***D–F***, Expression of *Lhx2* mRNA and mTmG (GFP) reporter in E12.5 control (***D***), *Emx1Cre;Lhx2^lox/lox^;mTmG* (***E***), and *NesCre;Lhx2^lox/lox^;mTmG* (***F***) brains. *Lhx2* is recombined and its expression is undetectable in the dorsal telencephalon of *Emx1Cre;Lhx2^lox/lox^;mTmG* brains, and mTmG reporter displays a complementary pattern, consistent with the activity domain for Emx1Cre (***E***). *NesCre;Lhx2^lox/lox^;mTmG* brains display no detectable Lhx2 expression and widespread expression of the mTmG reporter, consistent with the activity domain of NesCre (***F***). The control brains display autofluorescence in the green channel in the region of the choroid plexus which is an artifact. Scale bars: 100 μm.

Since early loss of LDB1 results in profound patterning defects, we used the later-acting Emx1Cre and NesCre lines to examine LDB1 function in the early development of the cortex and hippocampus. The Ai9 reporter was used to reveal the domain of Cre activity in *Emx1Cre;Ldb1^lox/lox^;Ai9* and *NesCre;Ldb1^lox/lox^;Ai9* embryos examined at E12.5. The Emx1Cre driver is known to recombine floxed alleles in the dorsal telencephalon starting from E10, and recombination is complete by E10.5 (Gorski et al., 2002; Shetty et al., 2013). Unexpectedly, a medio-lateral gradient of *Ldb1* expression was seen in the dorsal telencephalon of *Emx1Cre;Ldb1^lox/lox^;Ai9* embryos, suggesting that the floxed *Ldb1* alleles may not be completely recombined in the lateral telencephalon Fig. 2*B*, open arrowhead). We compared these results with embryos carrying the NesCre driver, which is known to be effective from E11.5 (Chen et al., 2015). In *NesCre;Ldb1^lox/lox^;Ai9*, we discovered that the floxed *Ldb1* appeared to be poorly recombined in the dorsal telencephalon by E12.5; however, the ventral telencephalon (vtel) and diencephalon (asterisks) displayed the expected loss of *Ldb1* expression. In contrast, the Ai9 reporter that was also present in each of these embryos displayed robust region-appropriate expression, restricted to the dorsal telencephalon in the case of Emx1Cre, and in the entire telencephalon and diencephalon in the case of NesCre.

Both the Emx1Cre and NesCre drivers are standard lines widely used in the literature. We tested whether they display the expected pattern of activity in another conditional line available to us that was homozygous for floxed *Lhx2* alleles (Mangale et al., 2008) together with a GFP reporter line (“mTmG,” Muzumdar et al., 2007). Both these loci displayed the expected spatial recombination pattern: *Lhx2* expression was undetectable in the dorsal telencephalon of *Emx1Cre;Lhx2^lox/lox^;mTmG* embryos at E12.5, and reporter GFP expression was robust in the same region; the ventral telencephalon and diencephalon displayed no apparent recombination of either the *Lhx2* or the mTmG locus. Likewise, *NesCre;Lhx2^lox/lox^;mTmG* embryos displayed complete *Lhx2* recombination and no detectable expression in the telencephalon or diencephalon, and GFP reporter expression in both structures. Together with the Ai9 data (Fig. 2*A–C*), this indicated that the Emx1Cre and NesCre drivers were working normally in our hands. For subsequent experiments, we used *Ldb1^lox/lox^* mice that did not carry any reporter.

**Figure 3.**
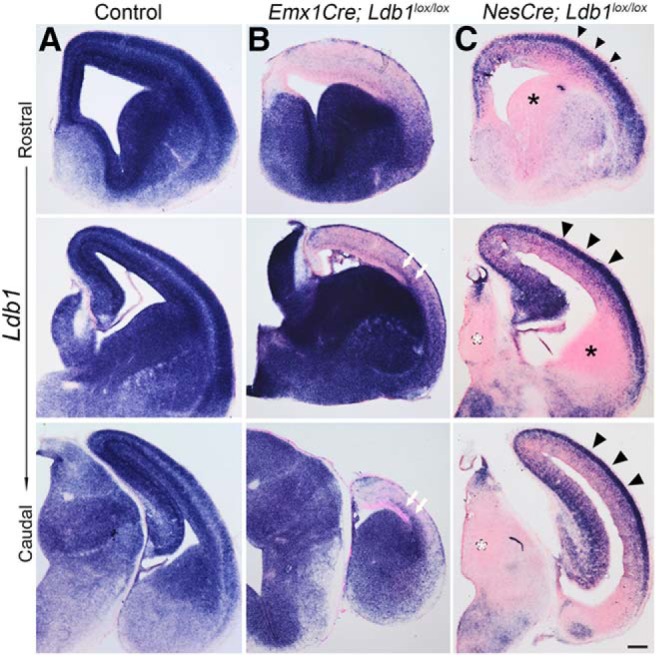
Recombination efficiency of the *Ldb1* locus improves with time in the case of Emx1Cre but not NesCre. ***A–C***, Expression of *Ldb1* mRNA in E15.5 control (***A***), *Emx1Cre;Ldb1^lox/lox^* (***B***), and *NesCre;Ldb1^lox/lox^* (***C***) brains at three rostro-caudal levels. *Emx1Cre;Ldb1^lox/lox^* brains display low *Ldb1* expression in the dorsal telencephalon, with no medio-lateral expression gradient. White arrows in ***B*** mark a spur of *Ldb1* expression consistent with the migrating interneuron stream that originates in the *Ldb1*-expressing ventral telencephalon. In contrast, *NesCre;Ldb1^lox/lox^* brains display robust expression in the cortical plate and in the hippocampus (black arrowheads), but the ganglionic eminences (black asterisks) and thalamus (white asterisks) appear to have lost *Ldb1* expression. Scale bars: 100 μm.

We tested whether the efficiency of recombination of the floxed *Ldb1* gene in the dorsal telencephalon improved with time. This appeared to be at least partially true for *Emx1Cre;Ldb1^lox/lox^* embryos, in which the recombination in the dorsal telencephalon appeared to improve by E15.5 at all rostro-caudal levels of sectioning. Only the lateral-most portion continued to display *Ldb1* expression (Fig. 3*B*, white arrows), which could be due to migrating interneurons from the *Ldb1*-expressing ventral telencephalon. In contrast, in *NesCre;Ldb1^lox/lox^*embryos, the neocortex and hippocampus continued to display robust *Ldb1* expression (Fig. 3*C*, black arrowheads), although the ganglionic eminences and thalamus appeared completely recombined (Fig. 3*C*, black and white asterisks, respectively).

In summary, in contrast to the Foxg1Cre driver which acts from E8.75, the *Ldb1* floxed allele appeared to be inefficiently recombined in the dorsal telencephalon by Cre drivers acting from E10.5 (Emx1Cre) and E11.5 (NesCre), and this did not ameliorate with time in the dorsal telencephalon in the case of the later acting driver NesCre. However, the efficient recombination of the *Ldb1* floxed locus in the developing thalamus of *NesCre;Ldb1^lox/lox^*embryos offered the opportunity of examining the role of LDB1 in the development of this structure without the complication of simultaneous loss of *Ldb1* in the neocortex. We therefore focused our attention on the diencephalon and thalamus.

**Figure 4.**
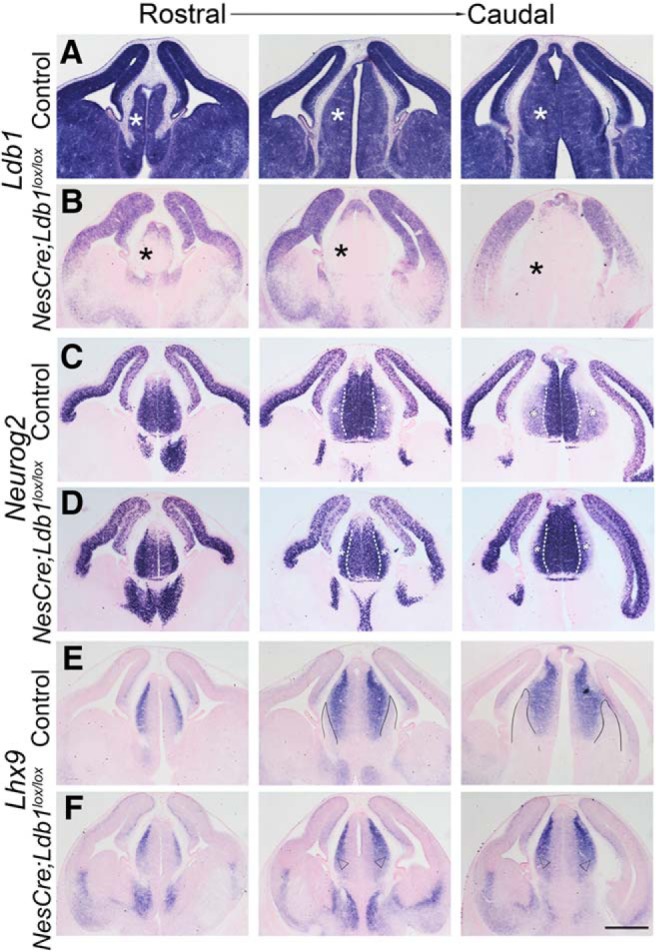
LDB1 is required for patterning the dorsal thalamus. ***A***, ***B***, Expression of *Ldb1* mRNA in E12.5 control (***A***) and *NesCre;Ldb1^lox/lox^* (***B***) brains at three rostro-caudal levels. In the mutant, *Ldb1* expression is undetectable in a broad region of the dorsal thalamus of the mutant brains (black asterisks, ***B***). ***C–F***, Serial sections from the same brains in ***A***, ***B*** probed for the expression of *Lhx9* and *Neurog2* at three rostro-caudal levels. In the mutant, the medial domain of intense *Neurog2* expression (white dashed lines, ***C***, ***D***) expands laterally at mid and caudal levels (white asterisks, ***C***, ***D***). At the same levels of sectioning, *Lhx9* expression, which is normally not seen in a lateral domain (solid black lines, ***E***), expands in the mutant to fill this domain (open arrowheads, ***F***). Scale bars: 500 μm.

First, we ascertained that the region of the diencephalon from which the thalamus arises did indeed lose *Ldb1* expression by E12.5. Indeed, in *NesCre;Ldb1^lox/lox^*embryos, *Ldb1* expression was undetectable in this region at all rostro-caudal levels (Fig. 4*B*, black asterisks), although expression in the dorsal telencephalon persisted. We examined the expression of *Neurog2,* which is enriched in progenitors (Fig. 4*C*, white dashed line), and the expression tapers off in lateral domains where postmitotic neurons reside. In the mutant embryos, *Neurog2* expression appeared to intensify in this lateral domain (Fig. 4*C*,*D*, white asterisks). We also examined *Lhx9* expression, which delineates three domains: expression is weak to undetectable in the medial progenitor domain, high in the adjacent domain containing postmitotic neurons, but undetectable in an extreme lateral domain (Fig. 4*E*, black lines). In the mutant diencephalon, *Lhx9* expression delineates only two domains, the medial progenitor domain lacking expression and a continuous lateral domain that displays expression (Fig. 4*E*,*F*, open arrowheads). The diencephalon appeared smaller in the mutant compared with control brains. Together, these results suggested a fundamental defect in diencephalic development on loss of LDB1.

**Figure 5.**
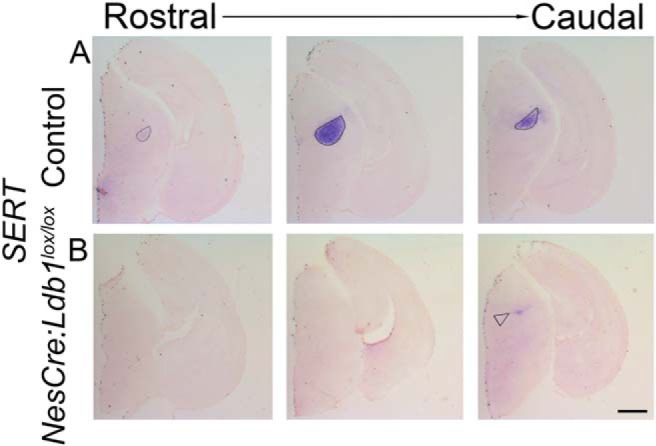
The somatosensory VB nucleus is profoundly shrunken on loss of LDB1. ***A***, ***B***, Expression of *SERT* mRNA in the VB nucleus in control (ovals, ***A***) and *NesCre;Ldb1^lox/lox^* (***B***) brains at three rostro-caudal levels. In the mutant, *SERT* expression is limited to a very narrow region in caudal sections, a severely diminished VB (open arrowhead, ***B***). Scale bars: 500 μm.

To explore the consequences of this early disruption of diencephalic development, we examined the thalamus of E17.5 *NesCre;Ldb1^lox/lox^*embryos with a battery of markers that are expressed in different sensory thalamic nuclei (Yuge et al., 2011). We first examined *SERT* expression, which identifies primary sensory nuclei (Lebrand et al., 1998). Whereas the control sections displayed intense *SERT* expression in the somatosensory ventrobasal (VB) nucleus (Fig. 5*A*, ovals), this nucleus appeared to be dramatically shrunken and was hard to identify in all but the most caudal levels of *NesCre;Ldb1^lox/lox^*brains (Fig. 5*B*, open arrowhead).

**Figure 6.**
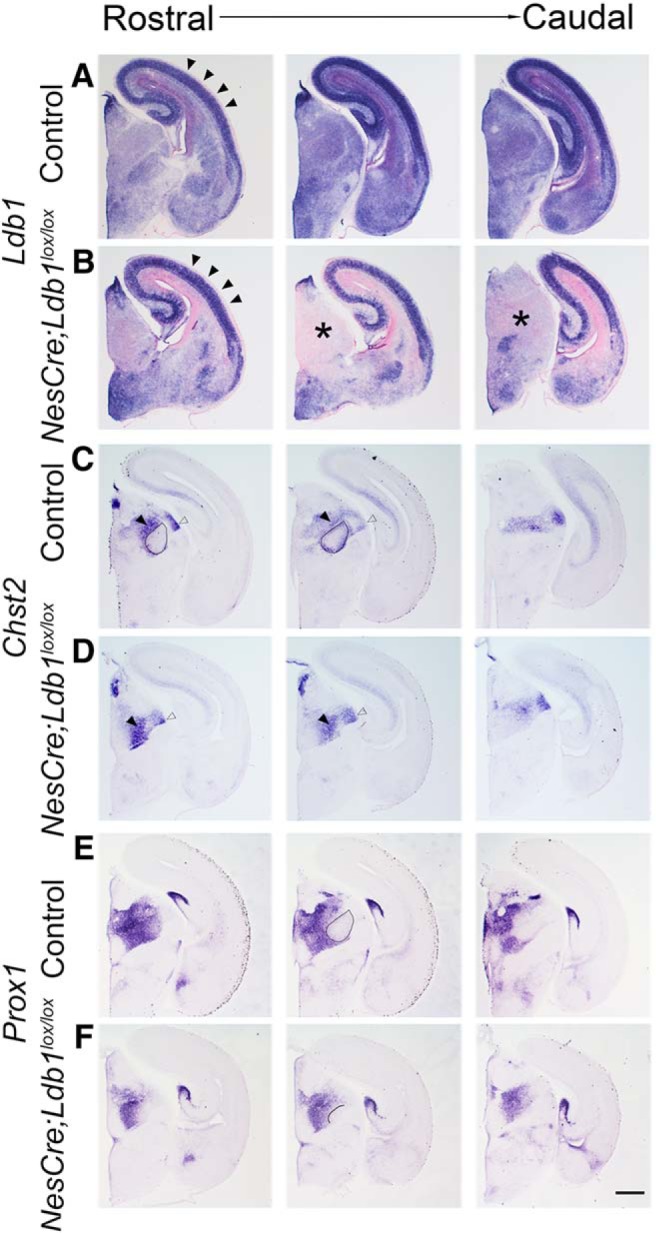
Multiple markers reveal shrinkage of the somatosensory VB on loss of LDB1. ***A–F***, Sections from E17.5 control (***A***, ***C***, ***E***) and *NesCre;Ldb1^lox/lox^* (***B***, ***D***, ***F***) embryos at three rostro-caudal levels. ***A***, ***B***, Expression of *Ldb1* mRNA in E17.5 control (***A***) and *NesCre;Ldb1lox/lox* (***B***) brains. In the mutant, *Ldb1* expression is undetectable in a broad region of the dorsal thalamus (asterisks, ***B***), while expression in the cortical plate (black arrowheads, ***A***, ***B***) and hippocampus persists. ***C–F***, *Chst2* expression is seen at the perimeter of the VB nucleus (ovals, ***C***), and *Prox1* expression excludes the VB nucleus leaving a distinct negative zone (oval, ***E***). These features are not revealed in mutant sections (***D***, ***F***), in which only a small *Prox1* negative domain is seen (solid line, ***F***). Black and open arrowheads identify the PO and the dLGN, respectively, which appear to be present in both control and mutant brains. Scale bars: 500 μm.

**Figure 7.**
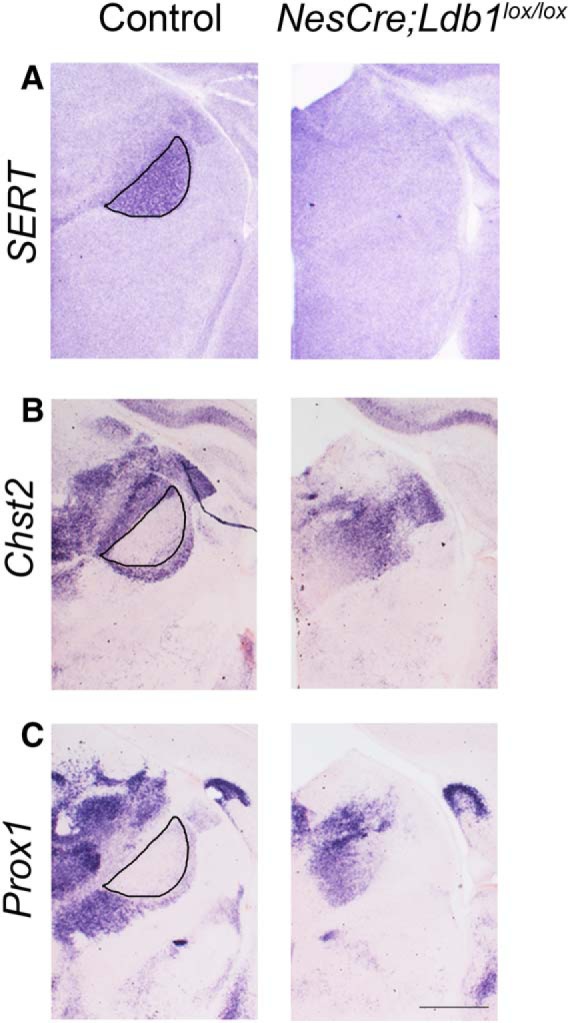
The VB is undetectable at P0 in LDB1 mutant brains. ***A–C***, Sections from P0 control and *NesCre;Ldb1^lox/lox^* brains. ***A***, *SERT* expression reveals the VB nucleus in control brains (black outline), which is undetectable in the mutant. ***B***, ***C***, *Chst2* and *Prox1* expression is seen at the perimeter of the VB nucleus in control (black outlines), but not in mutant brains. Scale bars: 500 μm.

We examined additional markers of the embryonic thalamus, *Chst2* and *Prox1*, to better understand the nature of the VB shrinkage. Since thalamic nuclei are small structures, we used one hemisphere of each brain to examine *Ldb1* expression, and the contralateral hemisphere to examine thalamic markers in serial sections. *Ldb1* expression was lost in the thalamus (asterisks) and persisted in the cortex (Fig. 6*A*,*B*, arrowheads). *Chst2* and *Prox1* expression delineates the border of the VB in control sections (Fig. 6*C*,*E*, ovals) but failed to exclude a territory consistent with the VB. However, *Chst2* expression was seen in the adjacent posteromedial thalamic nucleus (PO; solid arrowheads) and dorsal lateral geniculate nucleus (dLGN; open arrowheads) of both control and mutant brains, suggesting that the deficit may be specific to the VB (Fig. 6*C–F*). Unfortunately, the *NesCre;Ldb1^lox/lox^* mutants die at birth, so it was not possible to analyze the mutant thalamus at postnatal stages. However, we were able to harvest one mutant brain by monitoring the dam during delivery, and we examined adjacent sections of the thalamus for the expression of *SERT*, *Chst2*, and *Prox1* (Fig. 7). As at E17.5, it is not possible to identify the VB by *SERT* expression, and neither *Chst2* nor *Prox1* expression delineates this structure on loss of LDB1.

In summary, these data indicate that the VB critically requires LDB1 for its development and appears to be missing when *Ldb1* is disrupted in the thalamus using NesCre.

## Discussion

Using a conditional gene disruption strategy, we demonstrate for the first time the requirement of *Ldb1* in the development of the primary somatosensory VB nucleus of the thalamus. Since the neocortex displays strong *Ldb1* expression due to the apparent inability of NesCre to effectively delete the floxed *Ldb1* allele gene in this tissue, we had the fortuitous advantage of a system in which the thalamus, but not the cortex, lacks *Ldb1*. This allowed us to specifically examine any thalamic patterning defects that may have manifested independent of any cortex-derived influences; in any case, corticothalamic innervation does not enter the thalamus by E17.5 (Jacobs et al., 2007), making it more likely that the apparent loss of the VB is due to an autonomous requirement for LDB1. Furthermore, VB neurons are born at E11.5 (Angevine, 1970), so it is unlikely that the near-complete absence of the VB at E17.5 is due to a developmental delay in the production of these neurons. *Chst2* and *Prox1* expression appears to identify the adjacent PO, and *Chst2* also marks the dLGN in the mutant, indicating that the defect neither includes other somatosensory nuclei nor other primary sensory nuclei. This suggests that the VB may have a specific requirement for LDB1 for its development. Since such a defect has not been reported on loss of any known binding partners of LDB1 such as LHX2, LHX9, or NEUROG2 (Seibt et al., 2003; Lakhina et al., 2007; Marcos-Mondéjar et al., 2012), it remains an open question as to which factor(s) LDB1 complexes with to regulate VB development.

In contrast to the VB phenotype, the defects seen on early loss of LDB1 using Foxg1Cre appear to be consistent with a combination of the *Lhx2* and *Lhx5* mutant phenotypes. This is expected, since LDB1 is a known co-factor of LIM domain-containing transcription factors (Matthews and Visvader, 2003). The expansion of the hem and antihem seen on loss of LDB1 is similar to that reported in *Lhx2* null mutants (Bulchand et al., 2001; Mangale et al., 2008; Roy et al., 2013). Whereas *Lhx2* null embryos also have an expanded choroid plexus (Monuki et al., 2001), the complete absence of the choroid plexus on loss of LDB1 is similar to the phenotype reported for loss of *Lhx5* (Zhao et al., 1999). Loss of the choroid plexus is also seen in animals in which *Otx2* is disrupted either constitutively or conditionally (Matsuo et al., 1995; Johansson et al., 2013). OTX2 is known to act in a complex with LDB1 (Costello et al., 2015); therefore, the loss of the choroid plexus we report may be explained by the disruption of a complex containing LDB1 and either or both of LHX5 and OTX2.

Finally, it is an unexplained conundrum as to why the *Ldb1* locus, which is expressed in the entire forebrain throughout development (Bulchand et al., 2003), and therefore accessible in terms of the binding of transcriptional machinery, appears to be selectively resistant to recombination in the dorsal telencephalon from E10.5/E11.5, but not earlier. Very few studies have used the *Ldb1^lox/lox^* line to examine LDB1 function in the developing telencephalon. Zhao et al. (2014) used the Nkx2.1Cre driver and reported effective recombination in ventral telencephalic structures such as the medial ganglionic eminence (MGE) and the preoptic area (POA). This is consistent with our results in which we demonstrate that NesCre recombines the floxed *Ldb1* locus effectively in the ventral telencephalon. Leone et al. (2017) used the same Emx1Cre driver (Gorski et al., 2002) as in our study to inactivate *Ldb1* in the dorsal telencephalon. They report complete loss of *Ldb1* expression from the mutant cortex at postnatal day 4. We notice a progressive improvement of recombination such that the dorsal telencephalon is largely negative for *Ldb1* expression by E15.5, which is consistent with the findings of Leone et al. (2017). One study that reported incomplete *Ldb1* inactivation (Tzchori et al., 2009) found it to be due to lack of expression of the Cre driver in the cells that apparently escaped recombination (Narkis et al., 2012). In contrast, the Cre drivers in our study are expressed and effectively recombine other floxed lines (*Lhx2^lox/lox^*, mTmG, Ai9) in the dorsal telencephalon, but seem to be specifically inefficient at recombining the floxed *Ldb1* allele in this structure. One concern is that the presence of the Ai9 reporter could result in a competition for Cre recombinase, and the floxed *Ldb1* locus may not be favored for recombination over the reporter. However, except for Figure 2, none of the embryos used for the other figures carried the Ai9 reporter, so this does not explain the results. A mutation of the loxP sites could potentially make recombination of the *Ldb1* locus less likely. However, since *Ldb1* is efficiently recombined in the dorsal telencephalon by Foxg1Cre (Fig. 1), such a scenario is unlikely. Furthermore, any such mutation would have to arise only in the dorsal telencephalon, since the ventral telencephalon is efficiently recombined by NesCre. Another explanation could be that recombination does in fact take place, but existing *Ldb1* mRNA transcripts may persist due to the presence of RNA stabilizing proteins. The temporal regression of recombination efficiency, Foxg1Cre>Emx1Cre>NesCre, correlating with the timing of action of the Cre lines, could be explained by the progressive accumulation of such an RNA stabilizing protein specific to the dorsal telencephalon. Further studies are needed to examine these issues.

In summary, the incomplete recombination that we observe in the dorsal telencephalon seems to be a region-specific and temporally dynamic phenomenon that is unique to the *Ldb1* locus and has not been previously reported in any conditional mutant line. Our findings underscore the importance of examining the stage and extent of recombination when interpreting conditional loss of function phenotypes.

## Synthesis

Reviewing Editor: Esther Stoeckli, University of Zurich Decisions are customarily a result of the Reviewing Editor and the peer reviewers coming together and discussing their recommendations until a consensus is reached. When revisions are invited, a fact-based synthesis statement explaining their decision and outlining what is needed to prepare a revision will be listed below. The following reviewer(s) agreed to reveal their identity: Alexandre Dayer, Guillermina Lopez-Bendito.

Thank you for the revisions made on your manuscript. We have discussed the changes and came to the conclusion that we are satisfied with the revised version in most aspects. Obviously, a mechanistic explanation of the failure of Ldb1 recombination in the dorsal telencephalon would be desirable but we are aware that this would go beyond the scope of the manuscript. Still, we feel that reporting this observation is of interest to the community, as it calls for respective controls in many future studies. We would like to come back to one issue that we raised in response to your initial submission that was not addressed in your revision. The images reporting SERT expression are very misleading, as they suggest a broad expression of SERT in the mutant versus the control mice. You explain the difference in staining by 'overdevelopment' of the staining. However, we do not see a need for this. Please provide adequate images for this group, maybe along with sense controls.
